# A Novel Coarse Alignment Method for SINS Using Special Orthogonal Group Optimal Estimation

**DOI:** 10.3390/s20205740

**Published:** 2020-10-09

**Authors:** Fujun Pei, Su Yang, Desen Zhu, Shunan Yin

**Affiliations:** 1 Faculty of Information Technology, Beijing University of Technology, Beijing 100124, China; pfj@bjut.edu.cn (F.P.); zhudesen@bjtu.edu.cn (D.Z.); yinshunan@emails.bjut.edu.cn (S.Y.); 2 Engineering Research Center of Digital Community, Ministry of Education, Beijing 100124, China

**Keywords:** coarse alignment, special orthogonal group, swing base, optimal estimation

## Abstract

Aimed at the alignment problem of strapdown inertial navigation system (SINS) on the swing base, a novel coarse alignment method using special orthogonal group optimal estimation is proposed. There are two main contributions in this paper. First, based on the Lie group differential equation, the rotation matrix is updated directly by using error Lie algebra, which avoids the non-convexity of traditional methods and the need for non-collinear vector observation. Second is that a novel optimal estimation method is developed by using the exact error Lie algebra, which is calculated based on the physical definition of Lie algebra, as the innovation term to compensate the initial special orthogonal group in the estimation process. The asymptotic convergence of the proposed optimal estimation method is proved by Lyapunov’s second law. The simulation and experimental results demonstrate that the proposed method exhibits better performance than existing methods in alignment accuracy and time, which can achieve the self-alignment of SINS on the swing base.

## 1. Introduction

The strapdown inertial navigation system (SINS) is a completely independent navigation system that does not depend on the transmission of external signals [1], and it is therefore the most widely applied positioning and attitude determination navigation system [2]. The initial alignment is one of the key technologies of SINS and provides the initial attitude information for SINS. The accuracy of the initial alignment directly affects the navigation accuracy, and the duration time of the initial alignment impacts the system’s rapid response ability [3]. Since most of the current methods of self-alignment of SINS estimate the attitude matrix by both coarse and precise alignment [4,5,6], the accuracy and speed of coarse alignment directly influence the accuracy and speed of alignment.

Inertial navigation coarse alignment is divided into static and swing alignment according to the motion state of the carrier. Static coarse alignment uses gravity and the Earth’s rotation to determine the attitude matrix. At present, the problem of static coarse alignment has essentially been solved [3,7]. In the case of coarse alignment on the swing base, the carrier itself swings; for example, ships in mooring state swing because of engine speed, unit movement, cargo loading, wave impact, etc., and road vehicles swing because of engine speed, cargo loading, etc. Since the swing of the carrier itself makes it impossible to distinguish the angular velocity of the carrier from the angular velocity of the earth, the static alignment method cannot be used to determine the initial attitude matrix, and the noise will increase when the carrier shakes. Thus, it is necessary to design a new coarse alignment method that relies solely on gravity for observation and to minimize the impact of noise on the estimation results. 

Currently, the coarse alignment methods for SINS on a swing base can be divided into two categories: one category is to directly determine the initial attitude matrix through the gravity vectors of two or more moments by the TRIAD algorithm [8,9,10,11,12]. In [8], the initial rotation matrix is decomposed by attitude matrix decomposition to isolate the influence of the swing base and estimate the rotation matrix by two noncollinear gravity vectors. On this basis, [9,10,11] one can reduce the impact of the inertial measurement unit (IMU) output noise on the estimation results by adding a low-pass filter. Different from other studies [9,10,11], the authors of [12] used an LMS adaptive filter to reduce sensor noise, and the authors of [13] computed two initial rotation matrices using the gravity vectors from multiple moments and weighted averaged according to the standard deviation observed at each time, which improves the alignment accuracy. In an earlier study [14], to extract the effective observation vectors from the inertial sensors’ outputs, a method for parameter recognition and vector reconstruction was designed, where an adaptive Kalman filter was employed to estimate the unknown parameters. However, these alignment methods require two noncollinear gravity vectors as observations, which would require an interval of at least 50 s between each observation. Therefore, these methods require a long alignment time to ensure alignment accuracy. In addition, the insufficient utilization of observation information is another factor leading to low alignment accuracy. To solve this problem, researchers have proposed a second kind of method, which transforms the coarse alignment problem into Wahba’s problem [15,16,17,18,19,20]. In [15], the Q-method is suggested to resolve the Wahba problem to achieve coarse alignment. A method of extracting the attitude quaternion from the constructed K-matrix by the Newton iterative algorithm is proposed in [16]. In [17], the observation vector is recursively processed by using the filtered quaternion estimation (QUEST) method. An Optimal-REQUEST is proposed [18], and compared to the traditional attitude determination method, the filtering gain of the proposed method is tuned autonomously; thus, the convergence rate of the attitude determination is faster than in the traditional method. In contrast to the Q-method, a new method of resolving Wahba’s problem based on eigenvalues is recommended in [19], and symbolic solutions to the corresponding characteristic polynomial are derived. In an earlier study [20], a method for solving Wahba’s problem based on SVD decomposition is proposed. Although the above Wahba method does not require noncollinear vectors as observations, nonconvex issues and those regarding the fact that it is not guaranteed to be globally optimal occur in resolving Wahba’s problem.

In contrast to the quaternion, the special orthogonal group *SO*(3) conveniently represents the rotation in the serial movement of the carrier based on the mapping relationship between *SO*(3) and Lie algebra. Therefore, attitude estimation methods directly based on *SO*(3) have been developed instead of resolving Wahba’s problem in recent years. According to the properties and advantages of *SO*(3), an *SO*(3) optimization method is proposed in [21] by adopting the vector product of the measurement and prediction vectors as error Lie algebra and updating the rotation matrix based on the Lie group differential equation. Several cost functions are discussed in [22], which guarantee the properties of *SO*(3) in terms of the definition of error rotation, and the cost function itself is defined by the difference between the observation and prediction vectors. In [23,24,25], the Euclidean distance between the measurement and estimation vectors is employed as the attitude error function, and a system renewal equation is established with special orthogonal groups to realize attitude estimation. Although the abovementioned methods avoid the nonconvex problem of the Wahba methods and do not require noncollinear vectors as observation values, these methods all rely on the mapping relationship between the *SO*(3) group and Lie algebra as the update equation, and the innovation term applied in these estimation methods on *SO*(3) is not the true error of Lie algebra. Therefore, these existing estimation methods based on the special orthogonal group are not sufficiently accurate.

In this paper, a coarse alignment method based on the special orthogonal group optimal estimation method is developed. By integrating the advantages of inertial frame coarse alignment and special orthogonal group representation, a coarse alignment model is directly established based on the differential equation of the special orthogonal group. Because the state in the newly established coarse alignment model is the special orthogonal group of order 3 (SO(3)), a novel optimal estimation method based on the *SO*(3) group is developed to estimate the initial attitude. In this estimation method, based on the physical definition of Lie algebra, the solution of error Lie algebra is transformed into the calculation of the rotation axis and angle of the error rotation matrix to acquire the true error Lie algebra. According to the right-hand rule, the rotation axis is determined by the vector product of the prediction and observation vectors. The rotation angle is obtained by calculating the angle between the prediction and observation vectors. Then, the initial *SO*(3) group is compensated by using the true error Lie algebra as the innovation term based on the mapping relationship between the *SO*(3) group and Lie algebra. The Lyapunov stability analysis is employed to prove the stability of the novel optimal estimation method. Finally, simulation and experiments are designed to verify the performance with respect to the alignment accuracy and time, and the proposed method is compared to existing coarse alignment methods.

The organization of this paper is as follows: in the Section 2, the coarse alignment method based on *SO*(3) representation and optimal estimation is proposed. Section 3 analyzes the stability of the method developed in the second section. Section 4 provides the results of the simulation and contrast experiments, and Section 5 contains conclusions. The definitions of various reference systems in this paper are shown in Table 1

## 2. Coarse Alignment Model Using *SO*(3) Representation

In this section, to avoid the nonconvex issue when resolving Wahba’s problem and the long alignment time of the TRIAD algorithm, the initial alignment model based on *SO*(3) representation is established. The basics of Lie algebra and SO(3) are provided in the Appendix A.

According to the chain rule, the attitude matrix can be decomposed into the products of those attitude matrices corresponding to the motions of Earth and the body and the attitude matrix at the initial time. With *SO*(3) as the representation of the attitude, the attitude matrix on the swing base can be decomposed as follows:

(1) $$R_{b}^{n}\left(t\right)=R_{b\left(t\right)}^{n\left(t\right)}=R_{n\left(0\right)}^{n\left(t\right)}R_{b\left(0\right)}^{n\left(0\right)}R_{b\left(t\right)}^{b\left(0\right)}$$

where $R_{b\left(t\right)}^{n\left(t\right)}$ represents the attitude matrix of the current *b* frame relative to the current *n* frame, $R_{b\left(t\right)}^{b\left(0\right)}$ represents the attitude matrix of the current *b* frame relative to the initial *b* frame, $R_{b\left(0\right)}^{n\left(0\right)}$ represents the attitude matrix of the initial *b* frame relative to the initial *n* frame, and $R_{n\left(0\right)}^{n\left(t\right)}$ represents the attitude matrix of the initial *n* frame relative to the current *n* frame.

According to the differential equation of *SO*(3), the attitude matrix $R_{n\left(t\right)}^{n\left(0\right)}$ of the Earth’s motion and the attitude matrix $R_{b\left(0\right)}^{b\left(t\right)}$ of the body’s motion can be updated in the following ways:

(2) $${\dot{R}}_{n\left(t\right)}^{n\left(0\right)}=R_{n\left(t\right)}^{n\left(0\right)}(\omega_{in}^{n}\times )$$

(3) $${\dot{R}}_{b\left(t\right)}^{b\left(0\right)}=R_{b\left(t\right)}^{b\left(0\right)}(\omega_{ib}^{b}\times )$$

where $\omega_{ib}^{b}$ is the angular velocity of *b* frame relative to *i* frame under *b* frame, which can be measured by the gyro; $\omega_{in}^{n}=\omega_{ie}^{n}+\omega_{en}^{n}$, where $\omega_{ie}^{n}$ represents the angular velocity of *e* frame relative to *i* frame under *n* frame. The parameter $\omega_{en}^{n}$ is the angular velocity of *n* frame relative to *e* frame under *n* frame, and they can be calculated with the following equation:

$$\omega_{ie}^{n}=\left[\begin{array}{c} 0\\ \omega_{ie}\cos L\\ \omega_{ie}\sin L \end{array}\right],\;\;\;\;\;\;\;\omega_{en}^{n}=\left[\begin{array}{c} -\frac{V_{N}}{R_{M}+h}\\ \frac{V_{E}}{R_{N}+h}\\ \frac{V_{E}}{R_{N}+h}\tan L \end{array}\right]$$

where $R_{M}$ and $R_{N}\;$ are the radii of the curvatures of the meridian circle and prime unitary circle, respectively, of the carrier; $V_{N}$ is the speed along the north direction; and $V_{E}$ is the speed along the east direction. In the case of swing base, $V_{E}$ and $V_{N}$ are approximately equal to 0, so $\omega_{en}^{n}$ is approximately equal to 0; $\omega_{ie}$ is the rotation angular speed of Earth.

If the attitude matrix $R_{b\left(0\right)}^{n\left(0\right)}$ is estimated, $R_{b}^{n}\left(t\right)$ can be obtained by Equation (1). Therefore, the state equation is set as

(4) $$R\left(t_{k}\right)=R\left(t_{k-1}\right)$$

According to the alignment principles of SINS and the chain rule, the relationship between $V^{n}\left(t\right)$ and $V^{b}\left(t\right)$ is as follows:

(5) $$V^{n}\left(t\right)=R_{b\left(0\right)}^{n\left(0\right)}V^{b}\left(t\right)$$

where $V^{n}\left(t\right)$ and $V^{b}\left(t\right)$ are the velocities in *n* frame and velocity in *b* frame, respectively, and $V^{n}\left(t\right)$ and $V^{b}\left(t\right)$ can be obtained by the following equation:

(6) $$V^{n}\left(t\right)=-\int_{0}^{t}R_{n\left(t\right)}^{n\left(0\right)}g^{n}dt$$

(7) $$V^{b}\left(t\right)=\int_{0}^{t}R_{b\left(t\right)}^{b\left(0\right)}f^{b}dt$$

where $g^{n}={\left[\begin{array}{ccc} 0 & 0 & g \end{array}\right]}^{T}$ is the gravitational acceleration under *n* frame, g is the local gravitational acceleration, $g^{b}=-f^{b}$, $g^{b}$ is the gravitational acceleration relative to *b* frame, and $f^{b}$ can be measured by the accelerometer.

Therefore, the system model of coarse alignment based on *SO*(3) representation can be written as follows:

(8) $$\{\begin{array}{c} R\left(t_{k}\right)=R\left(t_{k-1}\right)\\ V^{n}\left(t\right)=R\left(t_{k}\right)V^{b}\left(t\right)\; \end{array}$$

## 3. Special Orthogonal Group Optimal Estimation

In this section, to avoid the error caused by using the inaccurate error Lie algebra as the innovation term in traditional *SO*(3) optimal estimation methods, a novel optimal estimation method on *SO*(3) is designed.

According to the definition of the rotation matrix and Equation (8), $V^{n}\left(t\right)$ and $R\left(t_{k}\right)V^{b}\left(t\right)$ are the same vectors under *n* frame, and the vector product of $V^{n}\left(t\right)$ and $R\left(t_{k}\right)V^{b}\left(t\right)$ is 0. However, due to the occurrence of errors, there is a deviation between the estimated and real rotation matrices $R\left(t_{k}\right)$, which causes the estimated rotation matrix $\hat{R}\left(t_{k}\right)$ to be the attitude matrix of *b* frame relative to $n^{\prime }$ frame.

(9) $$\hat{R}\left(t_{k}\right)=R_{b}^{n^{\prime }}\left(t_{k}\right)$$

**Figure 1:**
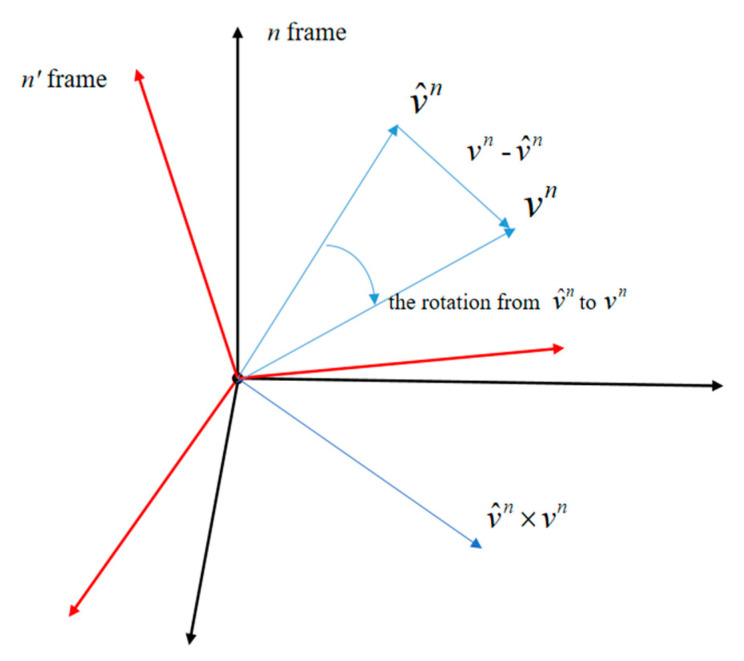
Diagram of the innovation vector.

According to the chain rule, to compensate for the estimated rotation matrix, we need to calculate the rotation relationship between the $n^{\prime }$ and n frames. According to the definition of coordinate system transformation, the above frame rotation relationship is equivalent to the rotation relationship between measurement vector $V^{n}\left(t\right)$ and prediction vector ${\hat{V}}^{n}\left(t\right)=\hat{R}\left(t_{k}\right)V^{b}\left(t\right)$, which has been previously described in a large number of papers [1,2,26]. According to the above conclusion, the existing *SO*(3) optimal estimation methods define the innovation as ${\hat{V}}^{n}\left(t\right)\times V^{n}\left(t\right)$ or $V^{n}\left(t\right)-{\hat{V}}^{n}\left(t\right)$, as shown in Figure 1.

**Figure 2:**
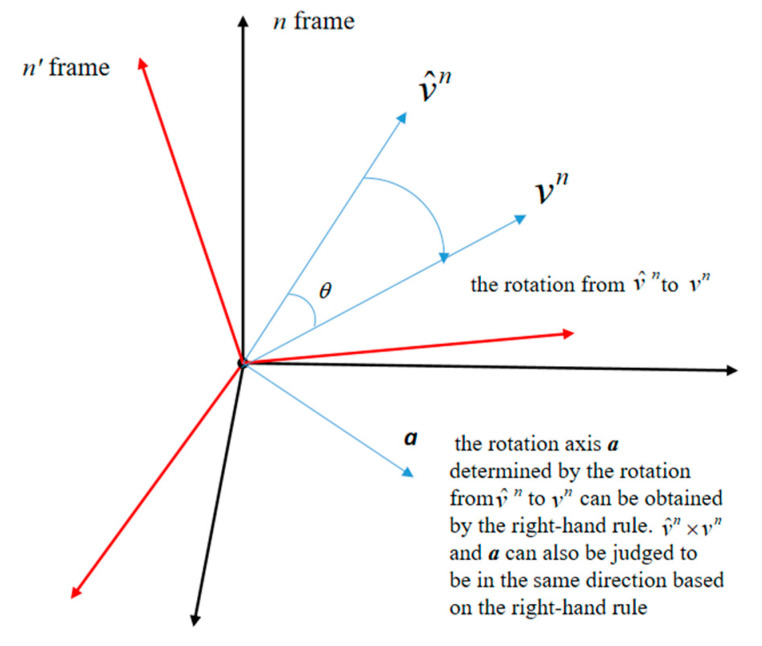
Schematic diagram of the rotation relation.

Because *SO*(3) optimization methods employ the mapping relationship between the SO(3) group and Lie algebra as the update equation, the innovation term should be the Lie algebra corresponding to the error rotation matrix. However, Figure 2 shows that these two vectors are not related to rotation, and although ${\hat{V}}^{n}\left(t\right)\times V^{n}\left(t\right)$ is oriented exactly in the same direction as the rotation axis, its length sum is obviously not equal to the error Lie algebra, while $V^{n}\left(t\right)-{\hat{V}}^{n}\left(t\right)$ is completely irrelevant to the error rotation matrix. Therefore, the innovation term used in the traditional *SO*(3) optimal estimation methods does not reflect the error of the attitude matrix, which inevitably affects the estimation accuracy.

According to the definition of Lie algebra (the rotation vector), it should be calculated as follows:

(10) l=θa

where a and θ are the rotation axis and angle, respectively, of the rotation relationship between $V^{n}\left(t\right)$ and ${\hat{V}}^{n}\left(t\right)$. Based on the right-hand rule, the rotation axis has the same direction as ${\hat{V}}^{n}\left(t\right)\times \;V^{n}\left(t\right)$, and the rotation angle θ is the angle between ${\hat{V}}^{n}\left(t\right)$ and $V^{n}\left(t\right)$, as shown in Figure 2.

Figure 2 reveals that ${\hat{V}}^{n}\left(t\right)\times V^{n}\left(t\right)$ exhibits precisely the same direction as the rotation axis a, and it can be calculated by unitizing ${\hat{V}}^{n}\left(t\right)\times V^{n}\left(t\right)$:

(11) $$a=\frac{{\hat{V}}^{n}\left(t\right)\times V^{n}\left(t\right)}{\left\|{\hat{V}}^{n}\left(t\right)\|\|V^{n}\left(t\right)\right\|}$$

Obviously, the rotation angle θ between ${\hat{V}}^{n}\left(t\right)$ and $V^{n}\left(t\right)$ is the angle between vectors ${\hat{V}}^{n}\left(t\right)$ and $V^{n}\left(t\right)$. Therefore, θ can be obtained by the following equation:

(12) $$\theta =\arcsin\frac{{\hat{V}}^{n}\left(t\right)\times V^{n}\left(t\right)}{\left\|{\hat{V}}^{n}\left(t\right)\|\|V^{n}\left(t\right)\right\|}$$

According to Equation (10), the accurate innovation term can be defined as:

(13) $${\tilde{y}}_{N}\left(t\right)=\theta a=\frac{{\hat{V}}^{n}\left(t\right)\times V^{n}\left(t\right)}{\left\|{\hat{V}}^{n}\left(t\right)\|\|V^{n}\left(t\right)\right\|}\arcsin\left(\frac{{\hat{V}}^{n}\left(t\right)\times V^{n}\left(t\right)}{\left\|{\hat{V}}^{n}\left(t\right)\|\|V^{n}\left(t\right)\right\|}\right)$$

Then, the update equation of *SO*(3) group optimal estimation can be established as follows:

(14) $$\dot{\hat{R}}\left(t\right)={\left[{\tilde{y}}_{N}\left(t\right)\right]}_{\times }\hat{R}\left(t\right)$$

## 4. Stability Analysis

The stability is one of the important properties of the control system, and stability analysis of the mathematical model based on the controlled object is an indispensable task in system design. For the proposed optimal estimation method, the purpose of stability analysis is to prove that the estimated value can approach the real value within a certain period of time. Therefore, Lyapunov stability analysis is conducted to prove that the proposed optimal estimation method is asymptotically stable.

The task of the optimal estimation method on *SO*(3) is to ensure that $\hat{R}\left(t\right)\to R\left(t\right)$ at time t→∞. Therefore, the system state error $\tilde{R}\left(t\right)$ is defined as follows:

(15) $$\tilde{R}\left(t\right)=\hat{R}\left(t\right)R{\left(t\right)}^{T}$$

According to the unit orthogonal property of the *SO*(3) group, when the estimated rotation matrix is equal to the real rotation matrix, the following applies:

(16) $$\tilde{R}\left(t\right)=I_{3\times 3}$$

Therefore, to prove the stability of the method proposed in this paper involves proving that $\tilde{R}\left(t\right)\to I_{3\times 3}$ at t→∞, which can be accomplished using Lyapunov’s second law.

Because $R^{T}$ is a time-invariant matrix, the derivative of the state error to time is as follows:

(17) $$\dot{\tilde{R}}\left(t\right)=\dot{\hat{R}}\left(t\right)R{\left(t\right)}^{T}$$

Substituting Equation (14) into Equation (17), we obtain

(18) $$\begin{array}{c} \dot{\tilde{R}}\left(t\right)=\dot{\hat{R}}\left(t\right)R{\left(t\right)}^{T}={\left[{\tilde{y}}_{N}\left(t\right)\right]}_{\times }\hat{R}\left(t\right)R{\left(t\right)}^{T}\\ ={\left[{\tilde{y}}_{N}\left(t\right)\right]}_{\times }\tilde{R}\left(t\right) \end{array}$$

The norm of the *SO*(3) matrix can be defined as follows [27]:

(19) $$\begin{array}{l} \|R{\left(t\right)\|}_{\mathrm{SO}\left(3\right)}={\langle \ln\left(R\left(t\right)\right),\ln\left(R\left(t\right)\right)\rangle }^{\frac{1}{2}}\\ ={\left[-\frac{1}{2}Tr\left(\ln\left(R\left(t\right)\right)\ln\left(R\left(t\right)\right)\right)\right]}^{\frac{1}{2}} \end{array}$$

For

(20) $$\ln\left(R\left(t\right)\right)=\Phi \left(t\right)=\phi {\left(t\right)}_{\times }$$

where 〈·,·〉 is the Lie bracket operation, and $\Phi \left(t\right)=\phi {\left(t\right)}_{\times }$ is the Lie algebra corresponding to the *SO*(3) group R(t). According to Equations (19) and (20), the candidate Lyapunov function is constructed as follows:

(21) $$W\left(\tilde{R}\left(t\right)\right)={\left(\|\tilde{R}{\left(t)\right\|}_{\mathrm{SO}\left(3\right)}\right)}^{2}=-\frac{1}{2}Tr\left(\tilde{\Phi }\left(t\right)\cdot \tilde{\Phi }\left(t\right)\right)$$

It can be proven that the above candidate Lyapunov function has the following properties:

**Property** **1.** *If and only if* $\tilde{R}\left(t\right)=I_{3\times 3},\;W\left(R\right)=0$ *; for any* $\tilde{R}\in SO\left(3\right)$ *and* $\tilde{R}\neq I_{3\times 3},\;W\left(\tilde{R}\right)>0$ *. In other words, the selected candidate Lyapunov function contains unique zeros and is positive definite.*

**Proof.**  (22) $$W\left(R\left(t\right)\right)={\left(||R{\left(t)|\right|}_{\mathrm{SO}\left(3\right)}\right)}^{2}=-\frac{1}{2}Tr\left(\tilde{\Phi }\left(t\right)\cdot \tilde{\Phi }\left(t\right)\right)$$

For any skew-symmetric matrix $\Phi =\left[\begin{array}{ccc} 0 & -\phi_{3} & \phi_{2}\\ \phi_{3} & 0 & -\phi_{1}\\ -\phi_{2} & \phi_{1} & 0 \end{array}\right]$, the following requirements are met:

(23) $$\begin{array}{c} Tr\left(\Phi \cdot \Phi \right)=Tr\left(\left[\begin{array}{ccc} 0 & -\phi_{3} & \phi_{2}\\ \phi_{3} & 0 & -\phi_{1}\\ -\phi_{2} & \phi_{1} & 0 \end{array}\right]\left[\begin{array}{ccc} 0 & -\phi_{3} & \phi_{2}\\ \phi_{3} & 0 & -\phi_{1}\\ -\phi_{2} & \phi_{1} & 0 \end{array}\right]\right)\\ =Tr\left(\left[\begin{array}{ccc} -\phi_{2}{}^{2}-\phi_{3}{}^{2} & \times & \times \\ \times & -\phi_{1}{}^{2}-\phi_{3}{}^{2} & \times \\ \times & \times & -\phi_{1}{}^{2}-\phi_{2}{}^{2} \end{array}\right]\right)\\ =-2\left(\phi_{1}{}^{2}+\phi_{2}{}^{2}+\phi_{3}{}^{2}\right)=-2\phi^{T}\phi \\ =-2{\left||\phi |\right|}^{2} \end{array}$$

Substituting Equation (23) into Equation (22), we obtain

(24) $$W\left(\tilde{R}\left(t\right)\right)=-\frac{1}{2}Tr\left(\tilde{\Phi }\left(t\right)\cdot \tilde{\Phi }\left(t\right)\right)={\left||\tilde{\phi }\left(t\right)|\right|}^{2}$$

When $\tilde{R}\left(t\right)=I_{3\times 3}$, the corresponding Lie algebra $\tilde{\phi }\left(t\right)=0$, for $W\left(\tilde{R}\left(t\right)\right)={\left||0|\right|}^{2}=0$, and when $\tilde{R}\left(t\right)\neq I_{3\times 3}$, the corresponding Lie algebra $\tilde{\phi }\left(t\right)\neq 0$, for $W\left(\tilde{R}\left(t\right)\right)={\left||\tilde{\phi }\left(t\right)|\right|}^{2}>0$. □

**Property** **2.** *For any* R(t)∈SO(3) *, function* $W\left(\tilde{R}\right)$ *is differentiable at time* t *, and* $\dot{W}\left(\left(\tilde{R}\right)\right)\leq 0$ *holds for* ∀t *. In other words, the selected candidate Lyapunov function is differentiable, and the derivative is negative.*

**Proof.**  (25) $$\begin{array}{l} \frac{d}{dt}W\left(\tilde{R}\left(t\right)\right)=-\frac{1}{2}Tr\left(\dot{\tilde{\Phi }}\left(t\right)\tilde{\Phi }\left(t\right)+\tilde{\Phi }\left(t\right)\dot{\tilde{\Phi }}\left(t\right)\right)\\ =-\frac{1}{2}Tr\left(\dot{\tilde{\Phi }}\left(t\right)\tilde{\Phi }\left(t\right)+{\left(\tilde{\Phi }\left(t\right)\dot{\tilde{\Phi }}\left(t\right)\right)}^{T}\right) \end{array}$$

For any matrix $M\in {\mathbb{R}}^{3\times 3},\;Tr\left(M+M^{T}\right)=Tr\left(M+M\right)$ holds, and then

(26) $$\begin{array}{c} \frac{d}{dt}W\left(\tilde{R}\left(t\right)\right)=-\frac{1}{2}Tr\left(\dot{\tilde{\Phi }}\left(t\right)\tilde{\Phi }\left(t\right)+{\left(\tilde{\Phi }\left(t\right)\dot{\tilde{\Phi }}\left(t\right)\right)}^{T}\right)\\ =-\frac{1}{2}Tr\left(\dot{\tilde{\Phi }}\left(t\right)\tilde{\Phi }\left(t\right)+\dot{\tilde{\Phi }}\left(t\right)\tilde{\Phi }\left(t\right)\right)=-Tr\left(\dot{\tilde{\Phi }}\left(t\right)\tilde{\Phi }\left(t\right)\right) \end{array}$$

To further prove that $\dot{W}\left(\tilde{R}\right)\leq 0\;$ holds for ∀t, the following theorems are required, which are proven in detail in [18]. □

**Theorem** **1.** *The derivative of Lie algebra*ϕ(t)*with respect to time has the following relation with its correspondingSO(3) group*R(t):

(27) $$\dot{\Phi }\left(t\right)={\left(Jr^{-1}\left(\phi \left(t\right)\right)SK^{-1}\left(\dot{R}R^{T}\right)\right)}_{\times }$$

**Theorem** **2.** *For any* $\;y\in {\mathbb{R}}^{3}\;$ *and* $u\in {\mathbb{R}}^{3},\;\Phi =\phi_{\times }$ *, the following equation holds:*

(28) $$-\frac{1}{2}Tr\left[{\left(Jr^{-1}\left(\phi \right)y\right)}_{\times }\Phi \right]=\phi^{T}y$$

Applying Theorem 1 to $\dot{W}\left(\tilde{R}\right)$:

(29) $$\dot{W}\left(\tilde{R}\left(t\right)\right)=-Tr\left[{\left[Jr^{-1}\left(\tilde{\phi }\left(t\right)\right)SK^{-1}\left(\dot{\tilde{R}}\left(t\right){\tilde{R}}^{T}\left(t\right)\right)\right]}_{\times }\tilde{\Phi }\left(t\right)\right]$$

Substituting Equation (18) into Equation (29) yields

(30) $$\dot{W}\left(\tilde{R}\left(t\right)\right)=-Tr\left[{\left(Jr^{-1}\left(\tilde{\phi }\left(t\right)\right){\tilde{y}}_{N}\left(t\right)\right)}_{\times }\tilde{\Phi }\left(t\right)\right]$$

Applying Theorem 2 to Equation (30):

(31) $$\dot{W}\left(\tilde{R}\left(t\right)\right)=2{\tilde{\phi }}^{T}\left(t\right){\tilde{y}}_{N}\left(t\right)$$

The positive and negative parts of Equation (33) are determined by the scalar product of the two vectors. Therefore, the positive and negative parts of Equation (31) can be determined by determining the angle between $\tilde{\phi }\left(t\right)$ and ${\tilde{y}}_{N}\left(t\right)$.

Because $\tilde{R}\left(t\right)$ is the Lie algebra corresponding to the error rotation matrix, the direction is the same as that of the rotation axis described by the error rotation matrix.

(32) $$\tilde{R}\left(t\right)=\hat{R}\left(t\right)R{\left(t\right)}^{T}=R_{b}^{n^{\prime }}\left(t\right)R_{n}^{b}\left(t\right)=R_{n}^{n^{\prime }}\left(t\right)$$

**Figure 3:**
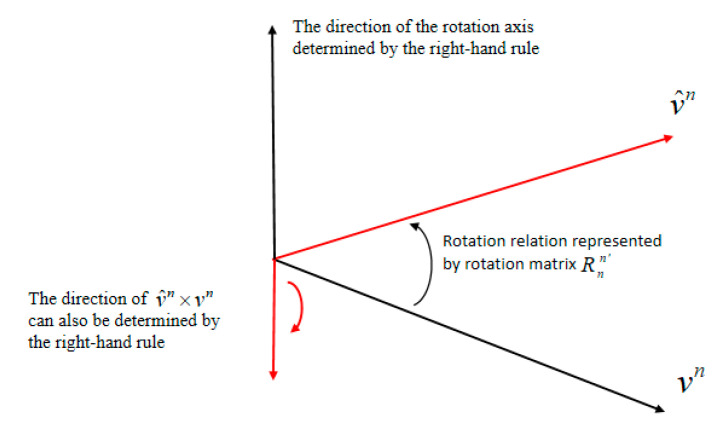
Schematic diagram of the rotation axis and error Lie algebra directions.

According to the definition of Lie algebra, the direction of $\tilde{\phi }\left(t\right)$ is the same as the direction of the rotation axis determined by $R_{n}^{n^{\prime }}$, as shown in Figure 3.

Equation (13) indicates that the direction of ${\tilde{y}}_{N}\left(t\right)$ is determined by ${\hat{V}}^{n}\left(t\right)\times V^{n}\left(t\right)$, and according to the right-hand rule, ${\hat{V}}^{n}\left(t\right)\times V^{n}\left(t\right)$ and the rotation axis determined by $R_{n}^{n^{\prime }}$ are oriented along the opposite direction. Therefore, ${\tilde{y}}_{N}\left(t\right)$ and $\tilde{\phi }\left(t\right)$ exhibit the opposite direction, and $\dot{W}\left(\tilde{R}\left(t\right)\right)=2{\tilde{\phi }}^{T}\left(t\right){\tilde{y}}_{N}\left(t\right)\leq 0$ holds. Due to the observation error, the direction of ${\tilde{y}}_{N}\left(t\right)$ may not be exactly opposite to that of $\tilde{\phi }\left(t\right)$, but as the observation error is not too large, $2{\tilde{\phi }}^{T}\left(t\right){\tilde{y}}_{N}\left(t\right)\leq 0$ can still be guaranteed.

In summary, the selected Lyapunov candidate function is positive definite and contains a unique zero point, and the derivative of the candidate function exists and is seminegative. Hence, the system is gradually stable.

## 5. Simulations and Experiments

In this section, simulations and experiments are performed to verify and test the performance and advantages of the special orthogonal group optimal estimation (SOGOE) proposed in this paper. In these simulations and experiments, the SOGOE method is compared to four currently popular coarse alignment methods, namely, the TRIAD method, the QUEST method, the adaptive identifier on special orthogonal group (AISOG) method and the fast-linear attitude estimator (FLAE).

The accuracy of these alignment methods is assessed by the difference between the true value of the attitude angle and that calculated by the estimated attitude matrix, as expressed in the following equation:

(33) $$\{\begin{array}{c} \delta \psi =\hat{\psi }-\psi \\ \delta \theta =\hat{\theta }-\theta \\ \delta \gamma =\hat{\gamma }-\gamma \end{array}$$

### 5.1. Simulation Results and Analysis

The simulation parameters and environment are as follows:

The equatorial radius is $R_{e}\;$= 6,378,165.0 m, the gravitational acceleration is *g* = 9.7849 m/s^2^, the angular velocity of Earth is 0.00007292758 rad/s, the initial position is east longitude 118°, and the north latitude is 40°. The gyro constant drift is 0.1°/h, the random drift is 0.01°/h, the constant deviation of the accelerometer is 1 × 10^−3^ g, and the random measurement noise is 3 × 10^−4^ g. The attitude update period is set as *T* = 0.02 s, and the alignment lasts 200 s.

The posture change trajectory is as follows:

(34) $$\{\begin{array}{c} \psi =20^{{}^{\circ}}+6^{{}^{\circ}}cos\left(\frac{\pi }{50}t\right)\\ \theta =5^{{}^{\circ}}+8^{{}^{\circ}}cos\left(\frac{\pi }{30}t\right)\\ \gamma =3^{{}^{\circ}}+4^{{}^{\circ}}cos\left(\frac{\pi }{20}t\right) \end{array}$$

where Ψ, θ and γ are the yaw, pitch and roll, respectively.

In the simulation experiment, Equation (34) is adopted as the real value of the attitude matrix, and the attitude angle errors are calculated with Equation (33).

**Figure 4:**
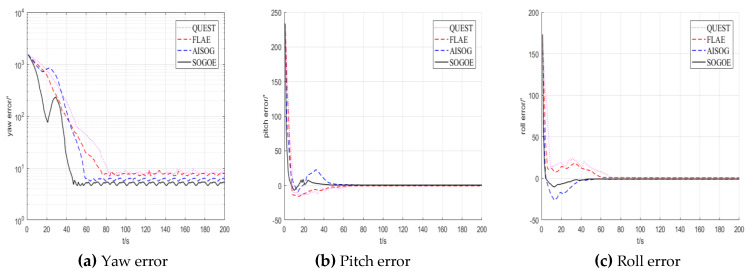
The attitude angle errors of the three alignment methods.

The alignment results of the simulation experiments are shown in Figure 4. The alignment time of the above three methods and the variance and mean analysis results of the four different methods from the beginning to the end of convergence are summarized in Table 2. In addition, the TRIAD algorithm is executed six times in total, each time requiring 100 s, and the mean value and variance in the six-time alignments with the TRIAD algorithm are also listed in Table 2. The standard deviations of the four different methods are provided in Table 3.

Figure 4 and Table 2 and Table 3 reveal that the alignment methods based on *SO*(3) optimization attain a better performance than the alignment methods based on the TRIAD algorithm and Wahba’s problem. More importantly, because the SOGOE method uses a more accurate error Lie algebra as compensation information, it is significantly better than the AISOG method in terms of the alignment speed and accuracy.

### 5.2. Experimental Results and Analysis

**Figure 5:**
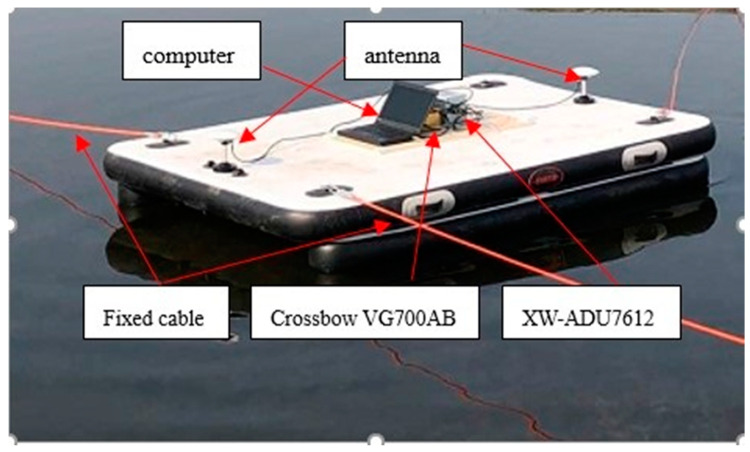
Installation diagram of the experimental facilities.

To verify the performance of the proposed coarse alignment based on SOGOE, we perform experiments on a swing base. The experimental equipment is installed on a surface platform, which is located on an open lake. In this experiment, the XW-ADU7612 Attitude Direction Unit Integrated Navigation System and Crossbow VG700AB are applied to conduct the experiments. XW-ADU7612 has a built-in high-precision IMU that independently determines north and is combined with a dual GPS, which outputs high-precision attitude information. In this experiment, the attitude information output of XW-ADU7612 is adopted as reference information. In addition, the XW-ADU7612 module operates stably, which makes the reference information it provides reliable. The system accuracy of this module is summarized in Table 4. In the experiment, Crossbow VG700AB is employed to obtain the values of the gyro and accelerometer. The precision of the IMU is provided in Table 5, and the installation of the experimental equipment is shown in Figure 5. During the experiment, we change the yaw by artificially pulling the platform and manually swing it to simulate the berthing of a ship on water. The initial position is 116.7536221°E and 40.0437500°N. The baseline length is 2.125 m. The computer used in the experiment has an I5 CPU, 16GB ram and 256GB hard disk to ensure the real-time performance. In addition, the computer can support multiple CPU cores to run real-time programs simultaneously. 

**Figure 6:**
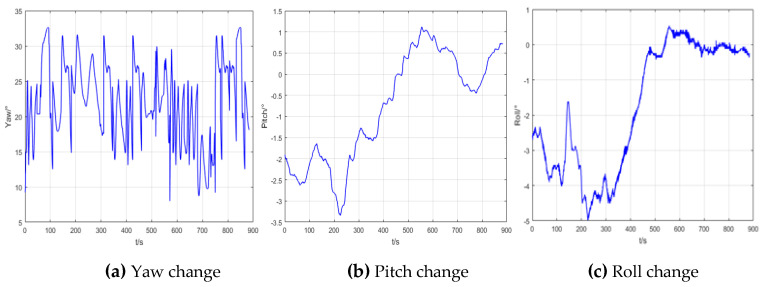
The change in attitude.

The change in the carrier attitude angle during the experiment is shown in Figure 6.

**Figure 7:**
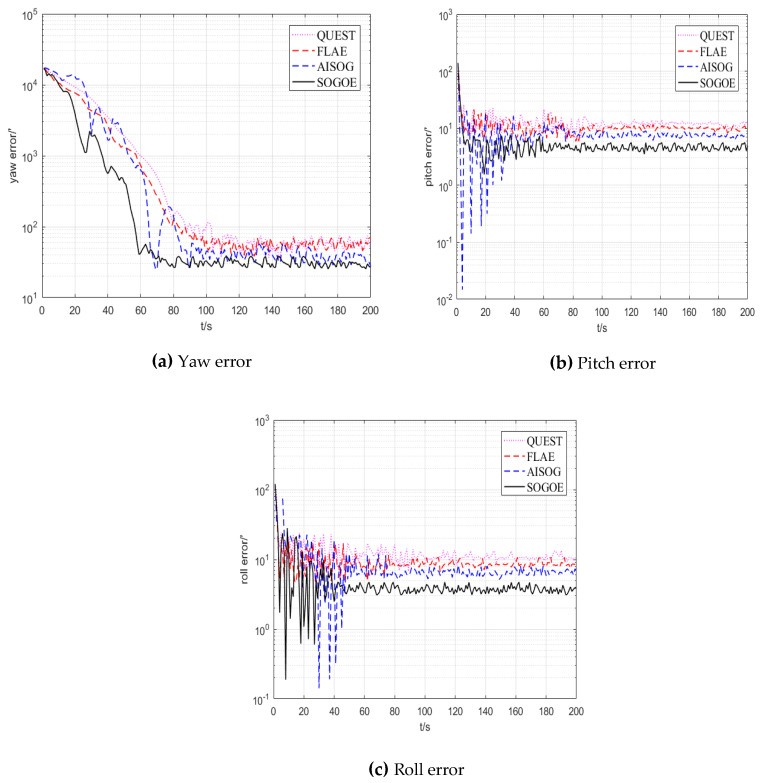
The attitude angle errors of the three alignment methods.

The alignment results of the experiment are shown in Figure 7. The alignment time of the above four methods and the variance and mean analysis results of the four different methods from the beginning to the end of convergence are summarized in Table 6. In addition, the TRIAD algorithm was executed six times in total, each time requiring 100 s, and the mean value and variance in the six-time alignments with the TRIAD algorithm are also provided in Table 6. The standard deviation of the three different methods is listed in Table 7.

As shown in Figure 7, since QUEST and FLAE are based on Wahba’s problem to estimate the initial rotation matrix, the above methods inevitably have the problem of non-convexity in Wahba’s problem, which leads to the poor accuracy and convergence speed of QUEST and FLAE compared with SOGOE and AISOG. More importantly, SOGOE attains higher estimation accuracy than AISOG because it uses a more accurate solution to error Lie algebra.

In order to better compare the results of the algorithm, the statistical results in Table 6 and Table 7 are used to further analyze the effect of the algorithm. It can be seen from Table 6 that TRIAD needs to use a pair of non-collinear vectors as observation, so its alignment time, variance and accuracy are poor. QUEST and FLAE can avoid the problem of triad by transforming the alignment problem into Wahba’s problem. However, the estimation accuracy and speed are limited due to the non-convex problem of Wahba’s problem. The optimization method based on *SO*(3) avoids the non-convex problem in Wahba’s problem and keeps the advantage of alignment speed based on optimization. It can be seen from Table 6 that the alignment accuracy and speed of AISOG are better than those of QUEST and FLAE. The most important thing is that SOGOE exerts better effect than AISOG by using a more accurate solution to error Lie algebra. Compared with the traditional QUEST and FLAE method, the alignment speed of SOGOE is improved by about 20 s, and the accuracy is improved by about 20′. In addition, since AISOG and SOGOE depend more on the accuracy of the observation information, the standard deviation increases with the increase in the observation noise under experimental conditions. However, it can be seen from Table 7 that the standard deviations of the two methods are still similar to those of QUEST and FLAE.

### 5.3. Position Estimation Experiment

**Figure 8:**
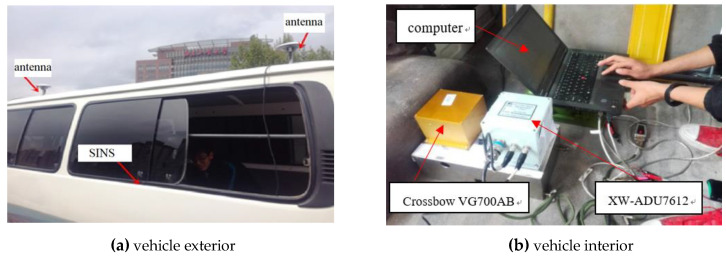
Installation diagram of the experimental facilities.

In order to reflect the influence of alignment results on the position estimation process, experiments are carried out on the actual road. This experiment also uses the XW-ADU7612 Attitude Direction Unit Integrated Navigation System and Crossbow VG700AB and the same computer to conduct the experiments. In this experiment, the attitude information output of XW-ADU7612 is adopted as reference information. The installation of the experimental equipment is shown in Figure 8. The antenna is installed on the roof of the car, with two antennas in the front and rear. The baseline length is 2.315 m. The inertial unit and the integrated navigation system are fixed in the car and coincide with the car body coordinate system.

**Figure 9:**
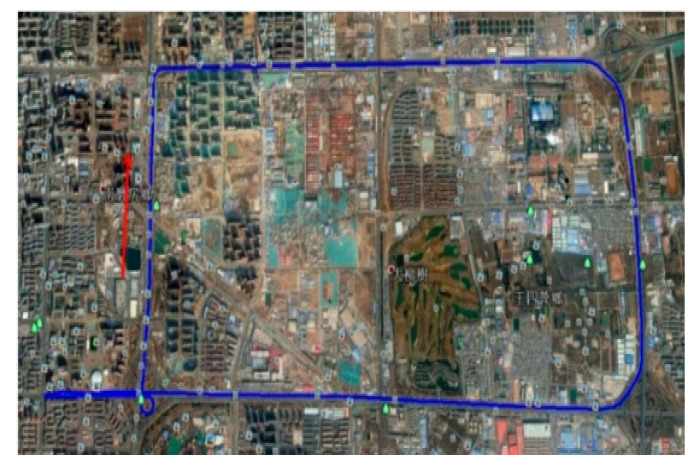
The vehicle trajectory.

**Figure 10:**
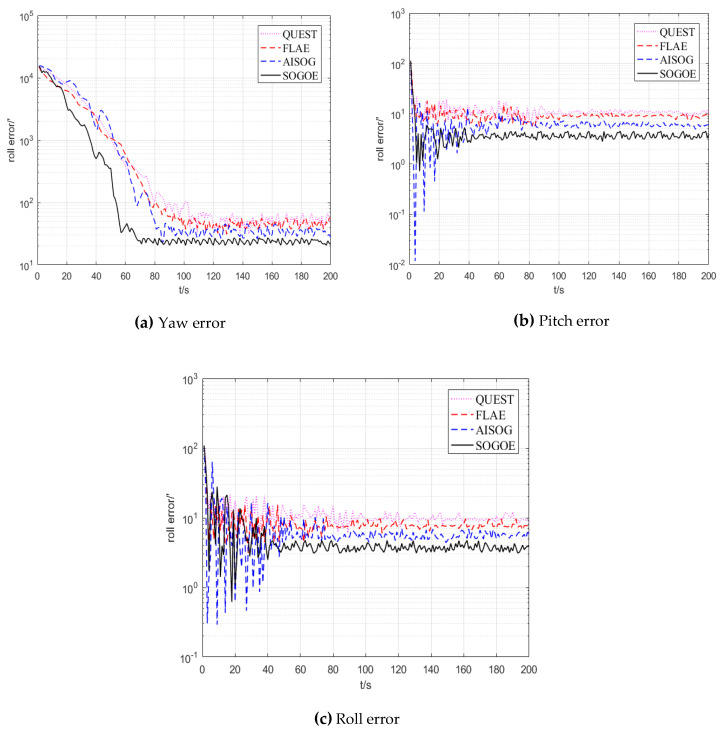
The attitude angle errors of the three alignment methods.

In order to ensure that the GPS receives good strength of signals during the experiment and that the experimental environment is sufficiently complex, the Fifth Ring Road in Beijing is selected as the test route, and the vehicle trajectory is shown in Figure 9. The alignment results of QUEST, FLAE, AISOG and SOGOE are shown in Figure 10 and Table 8.

**Figure 11:**
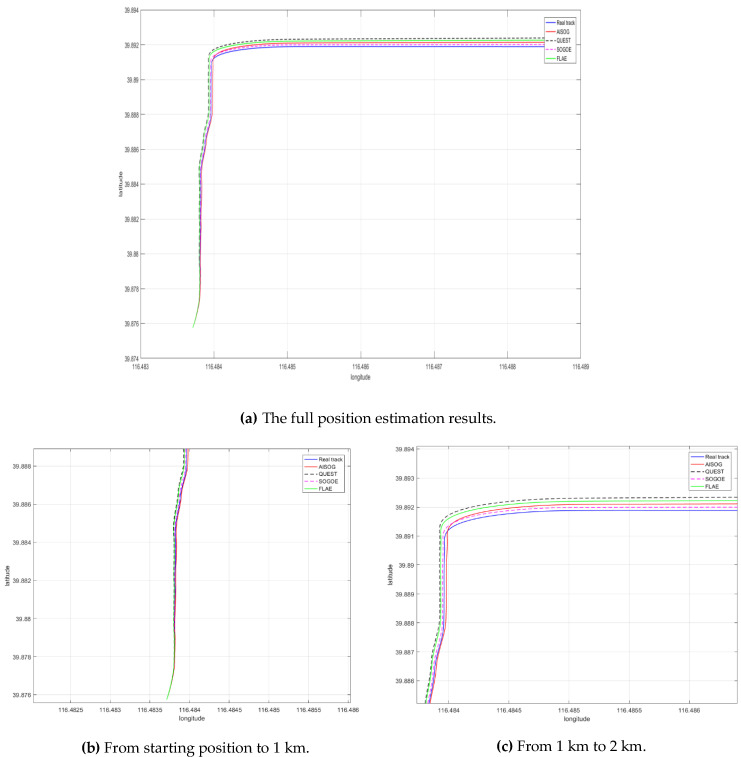
The position estimation results of the four alignment methods and real trajectory.

To illustrate the impact of alignment accuracy on position estimation, position calculations are performed based on the initial attitude matrices calculated by four methods. In order to highlight the influence of the initial rotation matrix on the position calculation, the position calculation only uses a simple method of integrating the IMU single sensor output to calculate the position. Since the location is solved in the above way, the result of long-term estimation is bound to diverge, so the position estimation results from the initial position to about 2000 m, where there is a significant deviation between the four methods and the real trajectories are intercepted, as shown in Figure 11.

As shown in Figure 11, due to alignment result errors, there is a deviation between the direction of speed and the actual course in subsequent position estimates. For a vehicle moving in a 2-D plane, this bias is mainly reflected in the heading bias in the position estimation process. Although the effect of alignment accuracy is not obvious when the driving distance is closer, the effect of initial alignment accuracy on position estimation increases as the driving distance increases. As shown in Figure 11, SOGOE has a significantly better alignment accuracy than other methods, so it can be seen that SOGOE is significantly better than traditional QUEST, FLAE, and AISOG methods when driving at around 1 km.

By comparing the simulation and experimental results of the five methods, it is found that the alignment method represented by *SO*(3) attains a higher alignment accuracy and shorter alignment time than the alignment method using the unit quaternion. Compared to AISOG, the SOGOE method proposed in this paper provides a more accurate update model to make the attitude estimation more accurate, which is especially evident in the experimental results. 

## 6. Conclusions

In this paper, a novel coarse alignment method using special orthogonal group optimal estimation is proposed for SINS on a swing base. The coarse alignment model is based on the Lie group differential equation of the special orthogonal group, which avoids the nonconvex problem of Wahba’s model in the traditional coarse alignment method. The proposed optimal estimation method for SO(3) adopts the precise error Lie algebra as the innovation term to compensate for the initial SO(3) group in the estimation process, which avoids the error caused by the inaccurate innovation term in traditional *SO*(3) optimization methods. Therefore, in theory, the proposed coarse alignment method has clear advantages in the alignment time and accuracy. The simulation and experimental results all prove that the proposed novel coarse alignment method demonstrates a clear improvement in the alignment time and accuracy over the existing methods. Therefore, Since investigations of *SO*(3) in inertial navigation are still relatively limited, further studies of the applications of *SO*(3) are planned.

Thus, the proposed method maintains the advantages of the attitude estimation method based on *SO*(3) optimization, and avoids the nonconvex issue of the coarse alignment problem based on Wahba’s problem which needs a non-collinear vector as observation. Moreover, the proposed optimal estimation method for *SO*(3) adopts the precise error Lie algebra as the innovation term to compensate for the initial *SO*(3) group in the estimation process. This avoids the error caused by the inaccurate innovation term in traditional *SO*(3) optimization methods. Although the proposed alignment method is more effective and exhibits excellent application prospects for SINS on the swing base, similar to most *SO*(3) optimization methods, the estimation results are more dependent on the accuracy of the observation information, and the variance of the estimation results will be greatly affected by the observation error.

## Figures and Tables

**Table 1:** The definition of the various reference frames in this paper.

Reference frame	Definition
***n* frame**	the navigation frame (*n* frame), which is the orthogonal reference frame aligned with east–north–up (E–N–U) geodetic axes
***b* frame**	the sensor body fixed frame
***i* frame**	the inertial coordinate frame
***e* frame**	the Earth frame
$n^{\prime }$ **frame**	the error *n* frame, obtained by the estimation method

**Table 2:** Comparison of the attitude angle error and alignment time.

		TRIAD	QUEST	FLAE	AISOG	SOGOE
**Yaw** **error (′)**	**Mean value**	11.6247	8.4124	7. 8728	5.7845	4.4568
	**Variance**	1.1421	0.8784	0.7925	0.5945	0.4254
**Pitch error (′)**	**Mean value**	1.2157	−0.6142	0.5852	0.5449	0.4246
	**Variance**	0.2463	0.0524	0.0513	0.0612	0.0386
**Roll** **error (′)**	**Mean value**	1.4245	0.8926	−0.8345	−0.7143	0.5218
	**Variance**	0.4662	0.0424	0.0413	0.0615	0.0325
**Alignment time/s**		110	82	76	65	45

**Table 3:** Standard deviation of the steady-state region.

STD	Yaw Error/′	Pitch Error/′	Roll Error/′
**QUEST**	0.937016	0.228975	0.205961
**FLAE**	0.890224	0.225831	0.202484
**AISOG**	0.771077	0.247507	0.247991
**SOGOE**	0.651920	0.196570	0.180388

**Table 4:** System accuracy of XW-ADU7612.

System Accuracy	
**Heading**	0.1° (baseline length > 2 m)
**Gyro zero-bias stability**	0.5°/h
**Accelerometer zero-bias stability**	≤1 mg

**Table 5:** System accuracy of Crossbow VG700AB.

System Accuracy		
	**Gyro**	0.5°/h
**IMU**	**Accelerometer**	≤1 mg
	**Data update rate**	100 Hz

**Table 6:** Comparison of the attitude angle error and alignment time.

		TRIAD	QUEST	FLAE	AISOG	SOGOE
**Yaw** **error (′)**	**Mean value**	89.6247	55.1124	51. 8728	43.5465	31.7568
	**Variance**	29.0471	8.2784	7.7925	9.6945	7.9254
**Pitch** **error (′)**	**Mean** **value**	19.1787	11.3142	10.2852	7.8249	4.3246
	**Variance**	4.1463	1.4314	1.3513	1.6435	1.42386
**Roll** **error (′)**	**Mean** **value**	18.6347	10.1426	8.8345	6.4312	3.8451
	**Variance**	3.4662	1.4254	1.2413	1.5495	1.26325
**Alignment time/s**		126	103	98	92	82

**Table 7:** Standard deviation of the steady-state region.

STD	Yaw Error/′	Pitch Error/′	Roll Error/′
**QUEST**	2.877221	1.196411	1.193901
**FLAE**	2.791504	1.162454	1.114136
**AISOG**	3.113599	1.281991	1.244789
**SOGOE**	2.815208	1.193256	1.123943

**Table 8:** Comparison of the attitude angle error and alignment time.

		QUEST	FLAE	AISOG	SOGOE
**Yaw** **error (′)**	**Mean value**	50.1124	46. 8728	38.5465	27.7568
	**Variance**	10.2784	9.7925	8.6945	6.9254
**Pitch** **error (′)**	**Mean** **value**	10.5142	9.2452	6.8249	3.3246
	**Variance**	1.7314	1.5513	1.2435	0.8386
**Roll** **error (′)**	**Mean** **value**	8.1426	7.8345	4.8312	3.2451
	**Variance**	1.4254	1.2413	0.9495	0.6325
**Alignment time/s**		100	95	84	74

## Appendix A

The basics of Lie algebra and SO(3) are provided in the following section.

A Lie group is a group ***G*** that is also a smooth manifold for which the group operations (g,h)→gh and $g\to g^{-1}$ are smooth. A Lie group is abelian if gh=hg for all g,h∈G. The special orthogonal group $SO\left(3\right)\times R^{3}$ is a subgroup of the general linear group GL(n), and the set of special orthogonal group is defined as

$$SO\left(3\right)=\left\{R:R\in {ℜ}^{3\times 3},R^{T}R=I_{3\times 3},det(R)=1\right\}$$

Its associated Lie algebra is a set of 3 × 3 skew-symmetric matrices:

$$SO\left(3\right)=\{A\in {ℜ}^{3\times 3}|A=A^{T}\}$$

The symbol (·×) represents the operation of converting a three-dimensional vector into a skew-symmetric matrix (SK(3)), where $\left[S_{\times }\right]$ is defined by

$$S_{\times }=\left[\begin{array}{ccc} 0 & -S_{3} & S_{2}\\ S_{3} & 0 & -S_{1}\\ -S_{2} & S_{1} & 0 \end{array}\right]\in {\mathbb{R}}^{3\times 3}$$

The inverse function is $\left[\cdot \right]\vee :so\left(3\right)\to S,S\in {ℜ}^{3}$.

$SK^{-1}()$ is the inverse operation of (·×).

$$SK^{-1}\left(S_{\times }\right)=S$$

Given R(t)∈SO(3), if matrix $R^{T}\left(t\right)\dot{R}\left(t\right)$ is skew-symmetric, then the differential equations of the Lie group satisfy the following:

$$\dot{R}\left(t\right)={\left[\omega \right]}_{\times }R\left(t\right)$$

where $\left[\omega \right]\times =R^{T}\left(t\right)\dot{R}\left(t\right)\in SO\left(3\right)$. For a given sampling time interval *T*, the discrete implementation of the differential equation is

$$\;R_{k+1}=R_{k}e^{T{\left[\omega \right]}_{\times }}$$

The matrix exponential that maps the Lie algebra ϕ (the rotation vector) onto the Lie group is well known and can be derived from Rodrigues’s equation for rotations.

R=exp(ϕ)=exp(θa)

$$=\left(\cos\theta \right)I+\left(1-\cos\theta \right)aa^{T}+\left(\sin\theta \right)a_{\times }$$

where a is the rotation axis, and θ is the angle of counterclockwise rotation about the rotation axis.

The initial alignment problem can be regarded as determining the attitude matrix between the carrier and navigation coordinate systems. Because the vectors in the attitude matrix A for SINS are orthogonal, the attitude matrix has the following properties:

$$AA^{T}=I,det(A)=1$$

According to the above definition of the Lie group, the attitude matrix only belongs to this group.
